# Evaluation of Pre-Treatment and Post-Treatment S100B, Oxidant and Antioxidant Capacity in Children with Diabetic Ketoacidosis

**DOI:** 10.4274/jcrpe.1716

**Published:** 2015-06-03

**Authors:** Cemil Kaya, Ali Ataş, Nurten Aksoy, Esra Celen Kaya, Mahmut Abuhandan

**Affiliations:** 1 Harran University Faculty of Medicine, Department of Pediatric, Şanlıurfa, Turkey; 2 Harran University Faculty of Medicine, Department of Pediatrics Endocrinology, Şanlıurfa, Turkey; 3 Harran University Faculty of Medicine, Department of Biochemistry, Şanlıurfa, Turkey; 4 Harran University Faculty of Medicine, Department of Physiotherapy, Şanlıurfa, Turkey

**Keywords:** diabetic ketoacidosis, S100B protein, oxidative state, antioxidative state

## Abstract

**Objective:**

The study aimed to evaluate the pre-treatment and post-treatment oxidant capacity, antioxidant capacity and S100B protein levels in cases of diabetic ketoacidosis (DKA).

**Methods:**

The study included 49 pediatric patients diagnosed with DKA and a control group comprising 49 healthy children. Blood samples were obtained after confirmation of the DKA diagnosis and also after treatment. S100B, total oxidant (TOL) and total antioxidant levels (TAL) were measured and the oxidative stress index (OSI) was calculated.

**Results:**

When the pre-treatment and post-treatment values of patients with DKA were compared with those of the healthy control group, the S100B level, TOL, TAL and OSI were found to be significantly higher in the diabetes group (p<0.001). Pre-treatment TOL and TAL were also significantly higher than post-treatment levels (p<0.001), while no statistically significant difference was found in the S100B levels or the OSI (p>0.05).

**Conclusion:**

We believe that long-term exposure to high blood glucose concentrations leads to an increase in TOL in patients with DKA and that the neurotransmitter changes that develop in response to this exposure lead to an increase in S100B levels, which is an indicator of neuronal damage.

## INTRODUCTION

Diabetes mellitus (DM) develops as a result of an actual or functional lack of insulin and it is a chronic condition that progresses to impairment in the patient’s carbohydrate, protein and lipid metabolism (1). The most common reason for the hospitalization of children with type 1 DM is diabetic ketoacidosis (DKA), which is also the leading cause of diabetes-related childhood mortality (2,3). Since constant glycemic control is not achieved in children during their decline to DKA, repetitive exposure to hyperglycemic and hypoglycemic episodes is common (4). Accordingly, such metabolic impairments have the potential to affect the developing brain (5). Neuropsychological studies performed on children with type 1 DM have shown adverse impacts on the brain (5,6).

S100B is a Ca-binding protein that is produced by astrocytes and has paracrine and autocrine effects on the neurons and the glia. S100B protein levels are thought to reflect pathologies in the brain and/or the blood-brain barrier and may be correlated with the severity of the damage to such an extent that they may be utilized as markers of damage (7). There are various studies defining the S100B protein as an easy-to-measure biological marker with an early prognostic value in the assessment of brain damage (8,9,10). Studies carried out in recent years indicate that the increased levels of free oxygen radicals and lipid peroxidation play a role in the pathogenesis of several disease states, many of which (including myocardial infarction, asthma, rheumatoid arthritis, cancer, stroke, epilepsy and a number of inflammatory disease states) have been reported to be associated with oxidative stress (11,12,13,14,15).

Studies have shown that the free radicals associated with hyperglycemia and the resultant oxidative stress are increased in children with DM and DKA (16,17,18,19). The purpose of the present study was to evaluate the S100B protein levels and oxidative state in children diagnosed with DKA.

## METHODS

### 

The study group comprised 49 children aged between 0.8-16.4 years, who were diagnosed with DKA based on ISPAD 2009 criteria (20) (plasma glucose >200 mg/dL, pH <7.3, serum bicarbonate <15 mEq/L, urine ketone ≥3+) and who were treated during hospitalization in the pediatric emergency service of the Harran University Faculty of Medicine (Şanlıurfa Province, Turkey) between October 2011 and February 2013. Patients who did not meet the criteria for DKA, those who had a history of afebrile convulsions, a metabolic disease, neurological sequelae and those diagnosed with degenerative and demyelinating diseases of the central nervous system as well as patients with cerebral edema were not included in the study. Another group of 49 healthy children aged between 0.6-16.0 years who visited the general pediatric outpatient clinic for purposes of immunization and/or health monitoring were included in the study as the control group.

Written consent was obtained from the parents of each child included in the study.

After obtaining a detailed medical history, each child underwent a physical examination. Based on the ISPAD 2009 protocol (20), appropriate liquids, insulin and other necessary treatments were scheduled and vital signs, blood glucose levels, complete blood, biochemistry and blood gas values were monitored closely in the DKA patients. After the dehydration and acidosis manifestations had improved and the patients regained consciousness, treatment and follow-up were continued with subcutaneous insulin and an appropriate diet. The study was approved by the Harran University Medical Faculty Ethics Committee. The families of the children included in the study were informed about the study and an informed consent form was signed.

Blood samples were obtained at the time of admission to the pediatric emergency clinic with diagnosis of DKA, before the initiation of iv insulin therapy. A second sample was obtained 2 hours after initiation of subcutaneous insulin therapy once the DKA presentation had improved. At the beginning of the study, a complete blood count was performed on the samples obtained from all patients and the healthy controls using an automated blood count device (Abbott Celldyn 3500 Ill, USA). Arterial blood gases were analyzed in the patients diagnosed with DKA using a Radiometer ABL800 device. Blood samples obtained from the study subjects were centrifuged for 5 minutes at 4000 rpm and the formed elements of the blood were discarded with the tube. The part of the serum sample that accumulated at the top was stored at -80 oC. Using the remaining serum samples, electrolyte levels, kidney and liver function tests were analyzed on the same day (Abbott Aeroset, Abbott Diagnostics, Abbott Park, IL, USA) and the serum samples stored at -80 oC were colorimetrically analyzed on the day of analysis with total oxidant levels (TOL) and total antioxidant levels (TAL) auto-analyzers (Abbott Aeroset, Abbott Diagnostics, Abbott Park, IL, USA) using the Erel method. Furthermore, S100B levels were also colorimetrically measured using the auto-analyzer (E-170 Roche®, Germany).

For estimation of S100B protein levels, S100B protein kits (Roche®, Germany) were used with a range of measurement of 0.005-0.105 µg/L. The analyses were carried out at the Harran University Medical Faculty Biochemistry Department Laboratories on the auto-analyzer device (E-170 Roche®, Germany), using the ECLIA (electrochemiluminescence) method.

TAL of the samples was analyzed using Rel assay commercial kits, following a method based on the reduction of all colored ABTS cationic radicals by the antioxidant substances available in the sample, which leads to a decolorization of the colored radicals proportionally to the total concentration of the antioxidant molecules. Trolox, a water-soluble analogue of the vitamin E, was used as a calibrator. The results are shown as mmoL Trolox-equivalent/L (21).

TOL was analyzed using Rel assay commercial kits. This method, as described in the working principle of the test, depends on a colorimetric measurement, in which the oxidant molecules in the sample oxidize cumulatively ferrous ions to ferric ions. The results are shown as μmoL H2O2 equivalent/L (22).

The oxidative stress index (OSI) is considered to be a marker of oxidative stress and is defined as the percent ratio of TOL to TAL. While calculating the OSI of the samples, TAL values are multiplied by 100 to equalize the units with the TOL (22). Results are shown as arbitrary units (AU).

### Statistical Analyses

The SPSS (Statistical Package for the Social Sciences, version 11.5 for Windows, SPSS® Inc, Chicago, IL) statistical analysis package was used. The distribution of the parameters was tested with a one-sample Kolmogorov-Smirnov test, from which it was confirmed that the distributions were good. Results were presented as mean ± standard deviation (SD). The comparison of the parameters of the patients and the control groups was made with independent-samples t-test and chi-square test. Pre-treatment and post-treatment values for the parameters associated with DKA were analyzed by the paired-samples t-test. A p-value smaller than 0.05 was considered statistically significant.

## RESULTS

Of the 49 enrolled patients with DKA, 28 (57.1%) were male and 21 (42.9%) were female. The mean age of the group was 9.5±4.8 years. Of the DKA patients, 21 (42.9%) had newly diagnosed type 1 DM, while 28 (57.1%) had been diagnosed with type 1 DM previously. Among the 49 subjects in the control group, 24 (49.0%) were male and 25 (51.0%) were female and their mean age was 8.9±4.3 years. The comparison of the age and gender distribution between the two groups revealed no statistically significant difference (p>0.05) (Table 1). Hemoglobin A1c (HbA1c), pH and HCO3 values at presentation in patients with DKA were 12.42±2.49, 7.12±0.15 and 9.67±4.18, respectively and the average duration of ketoacidosis was 13 hours (range: 5-120).

Pre-treatment mean TOL, TAL, OSI and S100B values of the DKA patients were significantly higher in the patient group as compared to the control group (respectively p<0.000, p<0.000, p=0.019 and p<0.000) (Table 1). A comparison p<0.000, p=0.007, p=0.001 and p<0.000) (Table 2). The pre-treatment mean TOL and TAL values of the DKA patients were also significantly higher than the post-treatment mean TOL and TAL values measured in the same group (respectively p<0.000 and p<0.001), whereas no statistically significant difference was detected in OSI or S100B levels (p>0.05) (Table 3).

Additionally, a positive correlation between S100B, total oxidant status and OSI was found (r=0.235, r=0.244, p=0.006 and p=0.005, respectively). S100B and total antioxidant status, pH at admission and HbA1c had no correlation. Also, no correlations between pH and HbA1c at admission and S100B, total antioxidant status, total oxidant status and OSI were found.

## DISCUSSION

Free radicals are continuously produced in biological systems (23). Recent studies have demonstrated that increased levels of free oxygen radicals and lipid peroxidation play a role in the pathogenesis of several diseases (11,12,13,14,15).

There is a relative scarcity of published reports examining oxidative markers and antioxidant levels in patients with DM or DKA. It was reported that oxidative stress is increased at a cellular level in patients with type 1 and type 2 DM when compared to controls (18), while another study noted that the oxidative stress associated with type 1 DM leads to repetitive DKA episodes and the development of fatal brain edema (16). Vantyghem et al (17) found that oxidative stress levels and prooxidant malondialdehyde levels are elevated in DKA patients. Brownlee et al (19) found that superoxide production during hyperglycemia plays a significant role in the pathogenesis of diabetic complications. In the present study, it was found that both the pre-treatment and post-treatment mean TOL values of the patients with DKA were significantly higher than those measured in the control group. Additionally, pre-treatment TOL values of the patients with DKA were significantly higher than post-treatment values. These findings can be associated with the increased production of free radicals and alterations in the antioxidant defense system due to non-enzymatic glycosylation, metabolic stress caused by changes in energy metabolism, sorbitol pathway activity and tissue damage that develops upon hypoxia and ischemia-reperfusion in DM and DKA (24). It is thought that the damage observed in the beta cells, known to be one of the most sensitive structures to oxidative stress, develops under the toxic effects of hyperglycemia. Rather that the DKA per se, which is an acute metabolic impairment, an increase in the oxidative stress observed during DKA may be associated with glucose instability in DM (17). Despite a decrease in TOL levels following treatment of DKA, they were still higher as compared to controls, suggesting that a certain degree of oxidative stress continues after correction of blood glucose levels and that elimination of oxidative radicals would require more time.

Substances that deactivate oxidants are called antioxidants. In normal healthy people, the free radicals and antioxidants are balanced, while in DM, this balance is impaired in favor of the free radicals (25,26). Previous studies have reported that the antioxidant enzymes are increased or decreased in cases of DM. In a study assessing such antioxidants as glutathione peroxidase (GPx), catalase and reduced glutathione (GSH) in patients with type 1 or type 2 DM, the levels of antioxidants in the DM group were found to be lower than those in the control group (18). Furthermore, in a study performed by Faure et al (27), the antioxidant levels measured in patients with DKA were also found to be lower than those in the control group. That said, there are contradictory studies reporting that the levels of an antioxidant may increase, remain the same, or decrease in DM (25,28,29,30). In the present study, both the pre-treatment and post-treatment mean TAL values of the patients with DKA were significantly higher than those of the control group. Additionally, mean pre-treatment TAL values of the patients with DKA were significantly higher than the mean post-treatment TAL values, which implies that this disease state activates a compensatory mechanism to protect the body from lipid peroxidation (28,30). In this case, it may be suggested that the level of antioxidants increases in order to neutralize the elevated levels of oxidants in patients with DKA and that this higher level is maintained in the post-treatment period.

There are only limited studies in the literature exploring the association between DKA and S100B and these studies have come up with contradictory results. In a case reported by McIntyre et al (31), S100B concentrations were shown to be elevated in a DKA patient with cerebral edema, a finding which led the authors to suggest that S100B can be a useful marker for the management of DKA. On the other hand, Roberts et al (32) reported that S100B is not elevated in DKA cases with cerebral edema. In the present study, we have found that both the pre-treatment and post-treatment mean S100B levels of patients with DKA are significantly higher than those of the control group. This finding may be associated with the decrease in extracellular GABA in relation to hyperglycemia, which results in an overall decrease in neuron inhibition and increased neuronal damage (7), as well as an increase in glutamate release and NMDA activity in patients with DM (33). The increased levels of glutamate result particularly in a Ca flow, which in turn increases the reactive oxygen species and causes a transmembrane ion imbalance. Correspondingly, a macromolecular flow is activated during which the free radicals cause structural and functional changes in the proteins within the neurons and in the glial cells and as a response to this oxidative action, the glial cells produce S100B. Additionally, the chronic hyperglycemia encountered during DKA can impair the neurotransmitter system and myelin production in a developing brain (34,35) and S100B levels may accordingly be increased in DKA patients. A comparison of the pre-treatment and post-treatment mean S100B levels of the patients revealed no statistically significant difference, implying ongoing neuronal degeneration due to the absence of normalization of oxidative radicals.

In our study, pre- and post-treatment OSI values in the patient group were higher than those of the controls. This finding may result from a relatively higher increase in total oxidant status than total antioxidant status, despite an increase in both variables as compared to controls. Pre- and post-treatment OSI did not differ significantly, due to a parallel increase in total oxidant status and total antioxidant status, precluding detection of statistically significant differences.

To the best of our knowledge, this study is the first highlighting the association between S100B, TAL and TOL in patients with DKA. It is obvious that further studies are required to confirm the results of the present study.

In conclusion, we believe that in patients with DKA, long-term exposure to high blood glucose concentrations during the development and course of the disease results in an increase in oxidative stress, while the dependently developing neurotransmitter changes cause an increase in the level of S100B, which is an indicator of neuronal damage. This situation underlines the significance of hyperglycemic control in patients with DKA.

## Figures and Tables

**Table 1:**
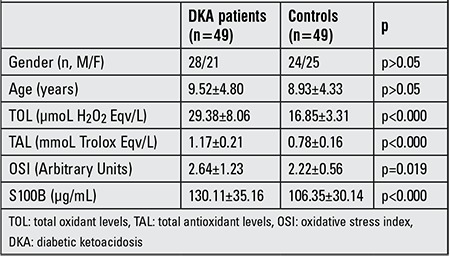
Demographic data and TOL, TAL, OSI, S100B levels in DKA patients (pre-treatment) and in the controls.

**Table 2:**
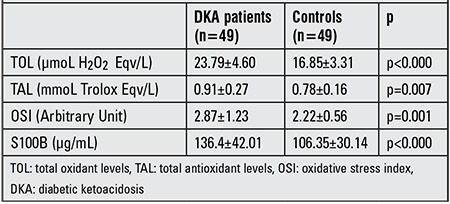
TOL, TAL, OSI and S100B levels in DKA patients (post-treatment) and in the controls.

**Table 3:**
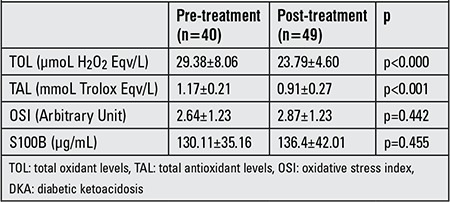
Pre-treatment and post-treatment TOL, TAL, OSI and S100B levels in DKA patients.
